# Hydrogen Sulfide Protects against Chemical Hypoxia-Induced Cytotoxicity and Inflammation in HaCaT Cells through Inhibition of ROS/NF-κB/COX-2 Pathway

**DOI:** 10.1371/journal.pone.0021971

**Published:** 2011-07-14

**Authors:** Chuntao Yang, Zhanli Yang, Meifen Zhang, Qi Dong, Xiuyu Wang, Aiping Lan, Fanqin Zeng, Peixi Chen, Chuhuai Wang, Jianqiang Feng

**Affiliations:** 1 Department of Physiology, Zhongshan School of Medicine, Sun Yat-sen University, Guangzhou, China; 2 School of Nursing, Sun Yat-sen University, Guangzhou, China; 3 Department of Physiology, Guangzhou Medical College, Guangzhou, China; 4 Department of Dermatology, Sun Yat-sen Memorial Hospital, Sun Yat-sen University, Guangzhou, China; 5 Department of Rehabilitation, The First Affiliated Hospital, Sun Yat-sen University, Guangzhou, China; Ohio State University, United States of America

## Abstract

Hydrogen sulfide (H_2_S) has been shown to protect against oxidative stress injury and inflammation in various hypoxia-induced insult models. However, it remains unknown whether H_2_S protects human skin keratinocytes (HaCaT cells) against chemical hypoxia-induced damage. In the current study, HaCaT cells were treated with cobalt chloride (CoCl_2_), a well known hypoxia mimetic agent, to establish a chemical hypoxia-induced cell injury model. Our findings showed that pretreatment of HaCaT cells with NaHS (a donor of H_2_S) for 30 min before exposure to CoCl_2_ for 24 h significantly attenuated CoCl_2_-induced injuries and inflammatory responses, evidenced by increases in cell viability and GSH level and decreases in ROS generation and secretions of IL-1β, IL-6 and IL-8. In addition, pretreatment with NaHS markedly reduced CoCl_2_-induced COX-2 overexpression and PGE_2_ secretion as well as intranuclear NF-κB p65 subunit accumulation (the central step of NF-κB activation). Similar to the protective effect of H_2_S, both NS-398 (a selective COX-2 inhibitor) and PDTC (a selective NF-κB inhibitor) depressed not only CoCl_2_-induced cytotoxicity, but also the secretions of IL-1β, IL-6 and IL-8. Importantly, PDTC obviously attenuated overexpression of COX-2 induced by CoCl_2_. Notably, NAC, a ROS scavenger, conferred a similar protective effect of H_2_S against CoCl_2_-induced insults and inflammatory responses. Taken together, the findings of the present study have demonstrated for the first time that H_2_S protects HaCaT cells against CoCl_2_-induced injuries and inflammatory responses through inhibition of ROS-activated NF-κB/COX-2 pathway.

## Introduction

Hydrogen sulfide (H_2_S), an endogenous gaseous mediator, is produced by pyridoxal-5′-phosphate-dependent enzymes, including cystathionine-γ-lyase (CGL, CSE), cystathionine-β-synthase (CBS) and 3-mercaptopyruvate sulfurtransferase (3-MST), during cysteine metabolism [1], [2]. Along with nitric oxide (NO) and carbon monoxide (CO), H_2_S is considered as the third signaling gasotransmitter, which plays important physiological and physiopathological roles both *in vivo* and *in vitro* [3], [4]. Accumulating evidence suggests that H_2_S exerts protective effects against various stimuli-triggered injuries in many organs including heart, liver and kidney [5], [6], [7]. One of the most important mechanisms responsible for H_2_S protection is antioxidation, which exerts its effect not only by increasing reduced glutathione (GSH) in neurons [8], but also by directly scavenging superoxide anions, hydrogen peroxide (H_2_O_2_) [9] and peroxynitrite [10] to suppress oxidative stress. The exact role of H_2_S in inflammation is controversial since both pro- and anti-inflammatory effects have been documented [11]. In sepsis, H_2_S provokes an inflammatory response via the extracellular signal-regulated kinase (ERK) pathway [12]. However, in lipopolysaccharide-stimulated microglias and astrocytes, H_2_S has an antiinflammatory effect [13]. To our knowledge, the role of H_2_S in hypoxia-caused dermatic injury has not been reported.

Hypoxia of skin is a common clinical event, which mediates dermatic injury in various diseases, such as pressure ulcer [14], diabetic ulcer [15], [16] and venous ulcer [17]. Insufficient blood or oxygen supply is considered as one of the most important causal factors, leading to non-healing chronic ulcers [18], [19], [20]. Overproduction of reactive oxygen species (ROS) caused by persistent hypoxia and disordered oxidative phosphorylation leads to dermatic injury. It has been demonstrated that pretreatment with the common antioxidant vitamin E significantly decreases pressure-induced skin lesions in pigs [21]. In addition, local administration of β-glucan suppresses skin injury by inhibiting malondialdehyde (MDA) production and raising GSH content [22]. The antioxidative effect of H_2_S has been demonstrated in a variety of cell models [8], [9], [10], [23]. Therefore, we hypothesize that H_2_S can also protect dermatic cells against oxidative stress-induced injury.

Inflammation is another mediator in dermatic injury induced by hypoxia. Cyclooxygenase (COX) and its catalysates, prostaglandins (PGs), are among the most important pro-inflammatory mediators. In chronic venous leg ulcers, COX-2 expression is upregulated and therefore responsible for persistent inflammation [24]. The selective inhibitors of COX-2 are effective in the treatment of this kind of disease. In addition, the protein complex nuclear factor kappa B (NF-κB) regulates inflammatory responses by inducing the expression of a variety of genes. NF-κB comprises a family of transcription factors, including the subunit members p50 (NF-κB1), p52 (NF-κB2), p65 (RelA), RelB and c-Rel [24]. Nuclear translocation of p65 subunit is a key step in the activation of NF-κB. In hypoxia-damaged HEI-OC1 mouse auditory cells, NF-κB and hypoxia-inducible factor-1 (HIF-1) are activated, thereby triggering interleukin-6 (IL-6) overproduction [25]. Our more recent study has demonstrated that chemical hypoxia induces inflammatory response and cytotoxicity through ROS-activated NF-κB/COX-2 pathway in human skin keratinocytes (HaCaT cells) [26]. However, it remains largely unknown whether H_2_S can abrogate this inflammatory response and cytotoxicity by inhibiting the ROS-activated NF-κB/COX-2 pathway in hypoxia-stimulated HaCaT cells.

In the present study, we investigated the cytoprotection of H_2_S in HaCaT cells treated with cobalt chloride (CoCl_2_), a well-known mimetic agent of hypoxia/ischemia, which induces oxidative stress [27], [28] and inflammation [29], [30]. HaCaT cells are derived from spontaneous transformation of human adult keratinocytes, and have been widely used in dermatopathological studies [31], [32]. Our findings showed that H_2_S protected HaCaT cells against CoCl_2_-induced injury and inflammatory response by inhibiting the ROS-activated NF-κB/COX-2 pathway.

## Materials and Methods

### Materials and cell culture

Sodium hydrosulfide (NaHS), CoCl_2_, N-acetyl-L-cysteine (NAC), pyrrolidine dithiocarbamate (PDTC), N-(2-cyclohexyloxy-4-nitrophenyl)-methane sulfonamide (NS-398) and 2′,7′-dichlorofluorescein diacetate (DCFH-DA) were purchased from Sigma-Aldrich (St Louis, MO). Cell Counter Kit-8 (CCK-8) was bought from Dojindo Laboratories (Kyushu, Japan). The GSH assay kit was obtained from Beyotime Institute of Biotechnology (Haimen, China). Enzyme-linked immunosorbent assay (ELISA) kits were provided by Boster BioTech. (Wuhan, China). Dulbecco's modified Eagle's medium F12 (DMEM/F12) and fetal bovine serum (FBS) were supplied by Gibco-BRL (Carlsbad, CA). HaCaT cells were generously provided by Professor Fanqin Zeng (Department of Dermatology, Sun Yat-sen Memorial Hospital, Sun Yat-sen University, Guangzhou, China) and maintained in DMEM/F12 supplemented with 10% FBS at 37°C under an atmosphere of 5% CO_2_ and 95% air.

### Cell viability assay

Cell viability was detected using CCK-8. HaCaT cells were cultured in 96-well plates, with 4 duplicate wells in each group. When 70–80% confluence was reached, the cells were treated with conditioned medium as indicated. The CCK-8 solution (10 µL) at a 1∶10 dilution with FBS-free DMEM/F12 (100 µL) was added to each well followed by a further 3 h incubation at 37°C. Absorbance was measured at 450 nm with a microplate reader (Molecular Devices, Sunnyvale, CA). The mean optical density (OD) of 4 wells in the indicated groups was used to calculate the percentage of cell viability as follows: percentage of cell viability = (OD_treatment group_−OD_blank group_)/(OD_control group_−OD_blank group_)×100%. The experiment was performed in triplicate.

### Measurement of inflammatory factors by ELISA

Secretions of IL-6, IL-8, IL-1β and prostaglandin E2 (PGE_2_) were determined by ELISA. HaCaT cells were plated in 96-well plates. After the cells were treated as indicated, the relative content of each secreted inflammatory factor in the supernatant was measured by ELISA according to the manufacturer's instructions (Boster BioTech, Wuhan, China). The relative content of the inflammatory factor in culture medium was normalized to cell viability. The experiment was carried out in triplicate.

### Western blot analysis

HaCaT cells were plated in 35 mm diameter petri dishes. When growing to 70–80% confluence, the cells were treated as indicated. At the end of treatments, HaCaT cells were harvested and resuspended in ice-cold cell lysis solution and the homogenate was centrifuged at 10,000×*g* for 15 min at 4°C. Total proteins in the supernatant were measured using a bicinchoninic acid (BCA) protein assay kit (Kangchen BioTech, Shanghai, China). Thirty micrograms of total proteins from each sample were separated by 12% sodium dodecyl sulphate–polyacrylamide gel electrophoresis (SDS-PAGE). The proteins in the gel were transferred into a polyvinylidene difluoride (PVDF) membrane. The membrane was blocked with 5% fat-free dry milk in TBS-T for 1 h at room temperature, and then incubated with the primary antibody specific to COX-1, COX-2, p65 subunit (Bioworld Technology, USA) or horseradish peroxidase (HRP)-conjugated β-actin (Kangchen BioTech, Shanghai, China) overnight with gentle agitation at 4°C. The next day, the membrane was washed and subsequently incubated with HRP-conjugated secondary antibodies for 1.5 h at room temperature. Following 3 washes with TBST, the membranes were developed using an enhanced chemiluminescence (ECL) kit (Applygen Technologies, Beijing, China) and exposed to X-ray films. Image J 1.41o software (National Institute of Health, Bethesda, MD, USA) was used to quantitatively analyze protein expression level.

### Measurement of ROS

Intracellular ROS content was determined by DCF staining followed by photofluorography. DCF is a fluorescent substance derived from cell-permeable DCFH-DA. HaCaT cells were cultured on a slide in DMEM-F12. DCFH-DA in FBS-free DMEM-F12 was added at a final concentration of 10 µM to the HaCaT cells. Cells were then incubated at 37°C for 30 min and the indicated treatments were performed. After all the treatments were accomplished, the slides were washed 3 times with FBS-free DMEM/F12, and DCF fluorescence was measured over the entire field of vision with a fluorescent microscope connected to an imaging system (BX50-FLA; Olympus, Tokyo). Mean fluorescence intensity, which represents the amount of intracellular ROS from 3 random fields, was analyzed using Image J 1.41o software.

### Measurement of GSH

The intracellular GSH content was measured using a commercially available kit (Beyotime Institute of Biotechnology, Haimen, China) as described previously [33]. The assay is based on the spectrophotometric measurement of 5-thio-2-nitrobenzoate (TNB), the product of a reaction with GSH. TNB was measured by detecting absorbance at 412 nm using a microplate reader. The obtained data were normalized to cell number. The experiment was carried out in triplicate.

### Statistics

All data were representative of experiments done in triplicate and were expressed as the mean ± standard error (SE). The assessment of differences between groups was analyzed by one-way ANOVA using SPSS 13.0 software. The differences between groups were compared with the least significant difference (LSD) test. Differences were considered significant if the probability (*P*)-value was <0.05.

## Results

### H_2_S inhibits CoCl_2_-induced cytotoxicity in HaCaT cells

**Figure 1:**
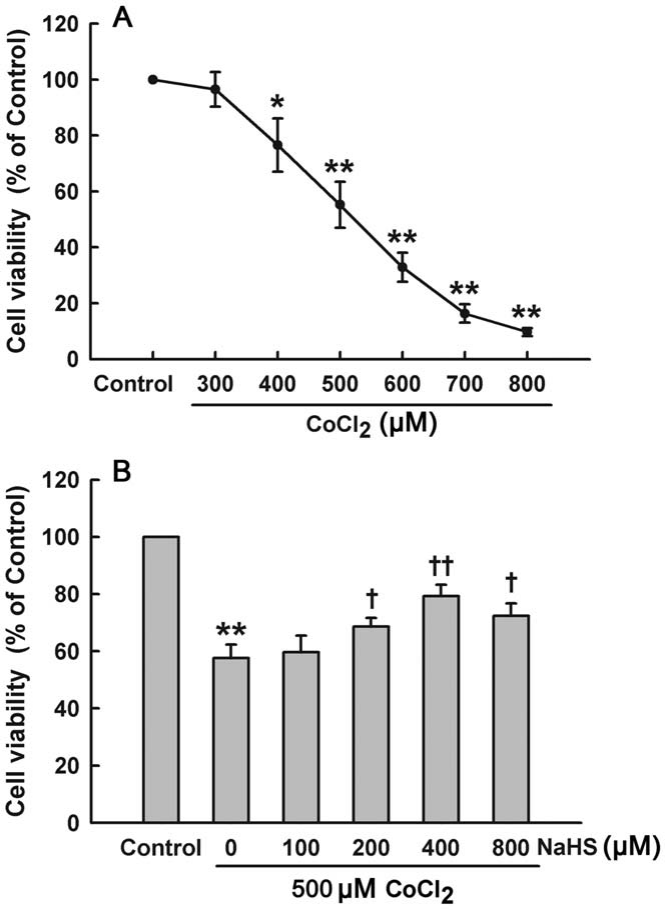
H_2_S protects HaCaT cells against CoCl_2_-elicited cytotoxicity. (**A**) HaCaT cells were treated with CoCl_2_ at indicated concentrations for 24 h. (**B**) Before exposure to 500 µM CoCl_2_ for 24 h, HaCaT cells were pretreated with different concentrations of NaHS for 30 min. Cell viability was measured by CCK-8 assay. Data were shown as the mean ± SE. ^*^ *P*<0.05, ^**^ *P*<0.01 compared with control group, ^+^ *P*<0.05, ^++^ *P*<0.01 compared with CoCl_2_ treatment group.

To investigate the effect of H_2_S on CoCl_2_-induced cytotoxicity, cell viability was detected by CCK-8 assay. As shown in Figure 1A, exposure of HaCaT cells to CoCl_2_ at concentrations ranging from 300 to 800 µM for 24 h led to a decrease in cell viability in a dose-dependant manner. However, the decreased cell viability induced by 500 µM CoCl_2_ treatment for 24 h was significantly inhibited by pretreatment with NaHS (a H_2_S donor) at 200, 400 or 800 µM for 30 min, respectively (Figure 1B). The results indicate that H_2_S pretreatment protects against CoCl_2_-induced toxicity in HaCaT cells.

### H_2_S ameliorates CoCl_2_-induced oxidative stress in HaCaT cells

**Figure 2:**
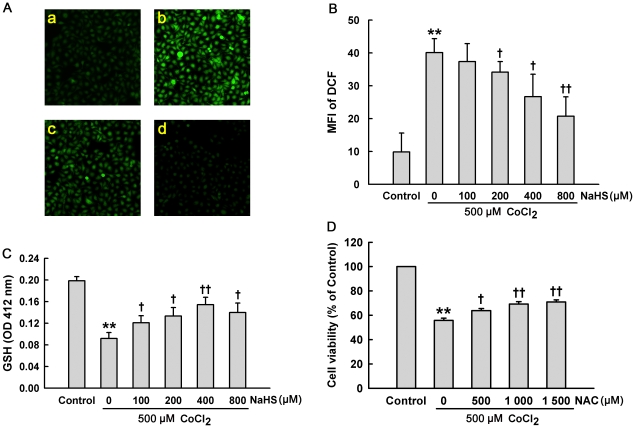
H_2_S reduces CoCl_2_-elicited oxidative stress in HaCaT cells. (**A**) a–d DCF staining followed by photofluorography to observe intracellular ROS level. (a) Control group. (b) HaCaT cells exposed to 500 µM CoCl_2_ for 2 h. (c) HaCaT cells were pretreated 400 µM NaHS for 30 min before exposure to CoCl_2_ at 500 µM for 2 h and (d) HaCaT cells were treated with 400 µM NaHS for 30 min followed by 2 h culture. (**B**) HaCaT cells were exposed to 500 µM CoCl_2_ for 2 h in the absence or presence of pre-incubation with the indicated concentrations of NaHS for 30 min. Quantitative analysis of the mean fluorescence intensity (MFI) of DCF with Image J 1.41o software. (**C**) HaCaT cells were exposed to 500 µM CoCl_2_ for 24 h in the absence or presence of pre-incubation with the indicated concentrations of NaHS for 30 min. The intensity of TNB (indicating GSH content) was measured at 412 nm with a microplate reader. (**D**) HaCaT cells were exposed to 500 µM CoCl_2_ for 24 h in the absence or presence of pre-incubation with NAC at the indicated concentrations for 60 min. Cell viability was measured by CCK-8 assay. Data were shown as the mean ± SE. ^**^ *P*<0.01 compared with control group. ^+^ *P*<0.05, ^++^ *P*<0.01 compared with CoCl_2_ treatment group.

To elucidate whether the cytoprotection of H_2_S was associated with its antioxidation in CoCl_2_-stimulated HaCaT cells, intracellular ROS and GSH levels were measured. Exposure of HaCaT cells to 500 µM CoCl_2_ for 2 h led to a noticeable increase in ROS level (Figure 2A-b and 2B). Prior to the CoCl_2_ exposure, pretreatment with NaHS at concentrations ranging from 100 to 800 µM for 30 min decreased intracellular ROS level in HaCaT cells (Figure 2A-c and 2B). Additionally, treatment of HaCaT cells with 500 µM CoCl_2_ for 24 h significantly decreased GSH level (Figure 2C), indicating that CoCl_2_ treatment impairs the endogenous antioxidant defense mechanism. Importantly, pretreatment with NaHS (100∼800 µM) obviously attenuated the inhibitory effect of CoCl_2_ on GSH level in HaCaT cells (Figure 2C). Further data showed that NAC, a common ROS scavenger, significantly attenuated the cytotoxicity induced by CoCl_2_ treatment in HaCaT cells (Figure 2D). These findings suggest that the inhibition of cytotoxicity of H_2_S is associated with its antioxidant effect.

### H_2_S represses CoCl_2_-induced inflammatory factor secretions from HaCaT cells

**Figure 3:**
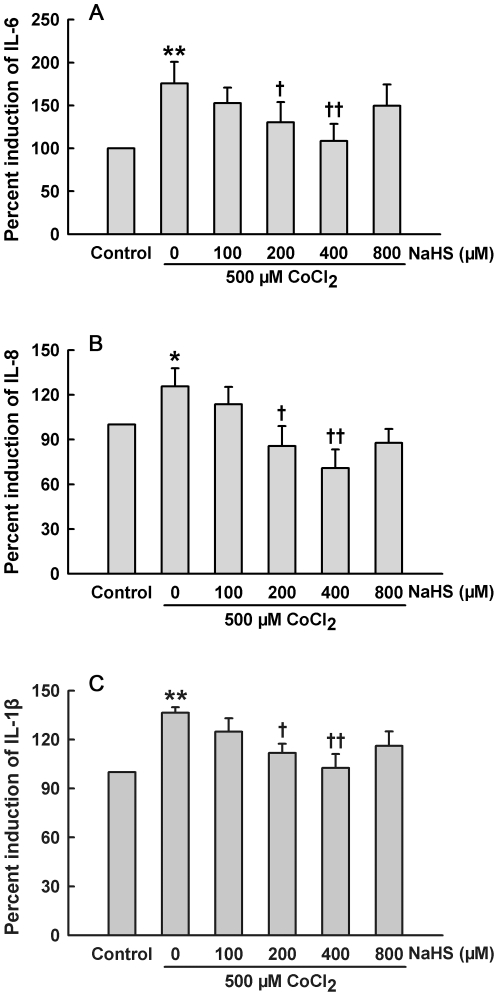
H_2_S inhibits CoCl_2_-induced IL-6, IL-8 and IL-1β secretions from HaCaT cells. HaCaT cells were exposed to 500 µM CoCl_2_ for 24 h in the absence or presence of pretreatment with NaHS at the indicated concentrations for 30 min. ELISA was performed to detect the levels of IL-6 (**A**), IL-8 (**B**) and IL-1β (**C**) in cell supernatants. Data were shown as the mean ± SE. ^*^ *P*<0.05, ^**^ *P*<0.01 compared with control group. ^+^ *P*<0.05, ^++^ *P*<0.01 compared with CoCl_2_ treatment group.

Next we measured IL-6, IL-8 and IL-1β secretions in response to NaHS and CoCl_2._ After exposure of HaCaT cells to 500 µM CoCl_2_ for 24 h, IL-6 (Figure 3A), IL-8 (Figure 3B) and IL-1β (Figure 3C) secretions were significantly increased, respectively. Pretreatment with NaHS (200 and 400 µM) for 30 min before exposure to CoCl_2_ markedly inhibited IL-6 (Figure 3A), IL-8 (Figure 3B) and IL-1β (Figure 3C) secretions from HaCaT cells, respectively. These results suggest that H_2_S possesses an anti-inflammatory effect in CoCl_2_-damaged HaCaT cells.

### Downregulation of COX-2/PGE_2_ overexpression contributes to the cytoprotection of H_2_S in CoCl_2_-stimulated HaCaT cells

**Figure 4:**
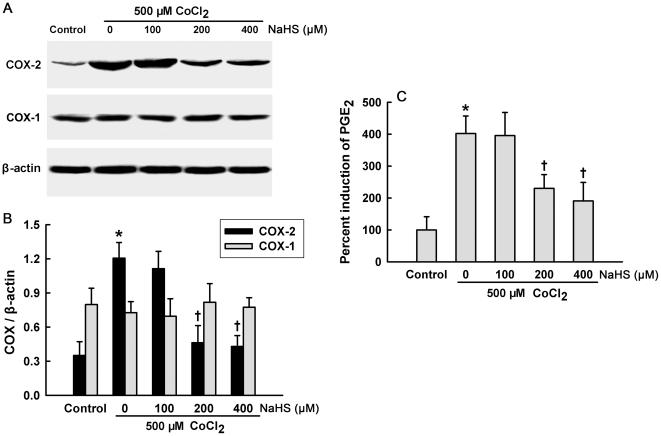
Effect of H_2_S on CoCl_2_-induced COX-2/PGE_2_ overexpression. HaCaT cells were incubated with 500 µM CoCl_2_ for 6 h in the absence or presence of pretreatment with NaHS at the indicated concentrations for 30 min. (**A**) Cell lysates were subjected to Western blot analysis using COX-2- or COX-1-specific antibody. (**B**) The intensity of the protein bands of a typical experiment was quantified with Image J 1.41o software. (**C**) PGE_2_ level in cell supernatants was measured by ELISA. Data were shown as the mean ± SE. ^*^ *P*<0.01 compared with control group. ^+^ *P*<0.01 compared with CoCl_2_ treatment group.

**Figure 5:**
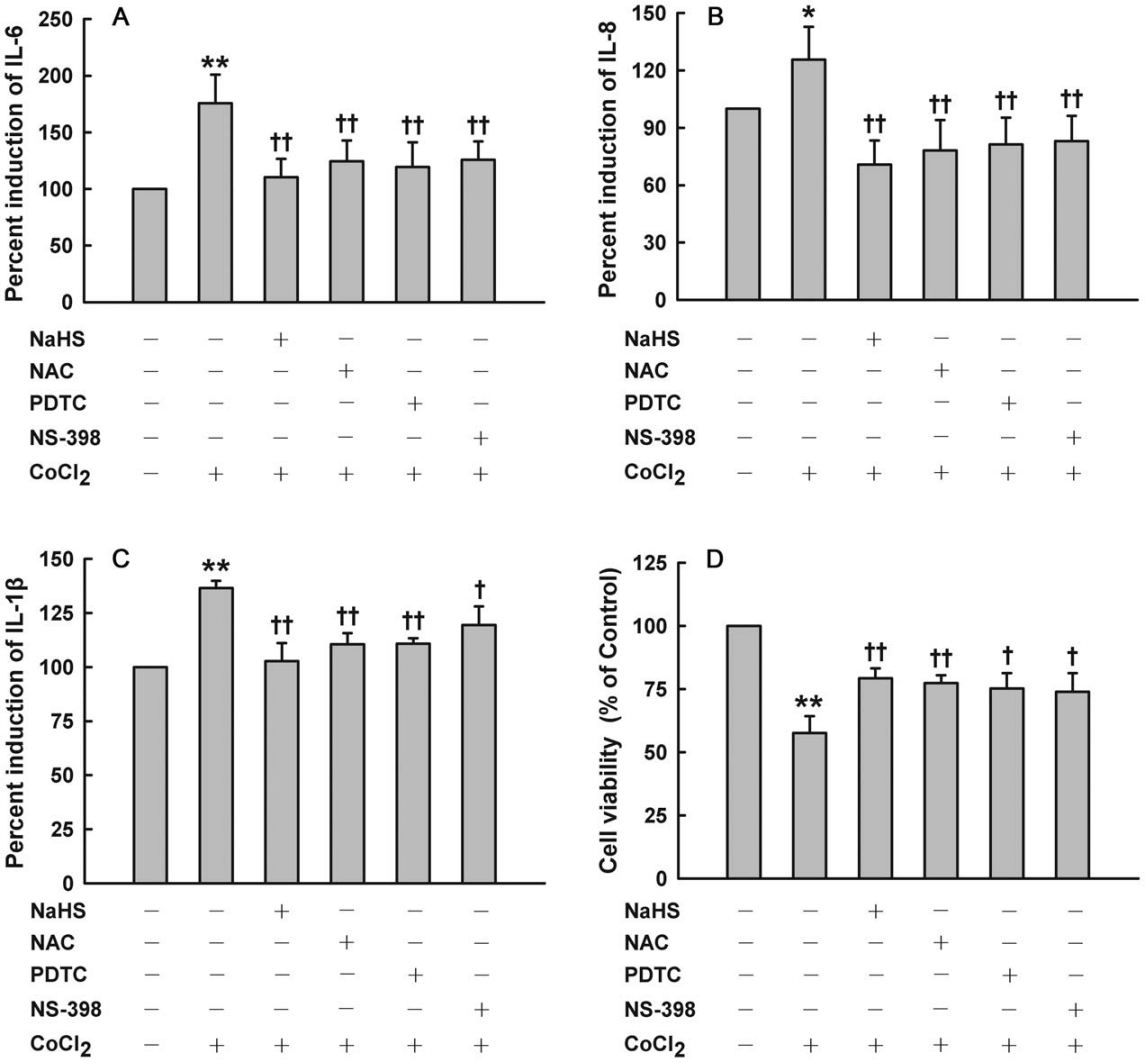
Effects of indicated treatments on secretions of IL-6, IL-8 and IL-1β secretions as well as cytotoxicity induced by CoCl_2_ in HaCaT cells. Before treatment with 500 µM CoCl_2_ for 24 h, HaCaT cells were pretreated with 400 µM NaHS, 10 µM PDTC or 10 µM NS-398 for 30 min, or 1000 µM NAC for 60 min, respectively. IL-6 (**A**), IL-8 (**B**) and IL-1β (**C**) secretions were detected by ELISA. (**D**) Cell viability was measured by CCK-8 assay. Data were shown as the mean ± SE. ^*^ *P*<0.05, ^**^ *P*<0.01 compared with control group. ^+^ *P*<0.05, ^++^ *P*<0.01 compared with CoCl_2_ treatment group.

After treatment of HaCaT cells with 500 µM CoCl_2_ for 6 h, expression of COX-2 was significantly augmented, while expression of COX-1 was not significantly changed (Figure 4A and B). Pretreatment with NaHS at 200 and 400 µM for 30 min markedly attenuated the overexpression of COX-2 induced by CoCl_2_ treatment (Figure 4A and B). Furthermore, exposure of HaCaT cells to 500 µM CoCl_2_ for 6 h resulted in an obvious increase in PGE_2_ secretion, which was blocked by pretreatment with NaHS at 200 and 400 µM for 30 min (Figure 4C). Additionally, pretreatment with NS-398, a selective inhibitor of COX-2, could imitate the roles of H_2_S in inhibition of inflammatory factor secretions, including IL-6 (Figure 5A), IL-8 (Figure 5B) and IL-1β (Figure 5C), as well as cytotoxicity (Figure 5D) induced by the CoCl_2_. These findings suggest that COX-2/PGE_2_ pathway mediates CoCl_2_-induced cytotoxicity and inflammatory response, and that the inhibition of CoCl_2_-induced COX-2/PGE_2_ overexpression is involved in the H_2_S-triggered protective effect in HaCaT cells.

### Inhibition of nuclear translocation of NF-κB p65 subunit is implicated in the cytoprotection of H_2_S in CoCl_2_-stimulated HaCaT cells

**Figure 6:**
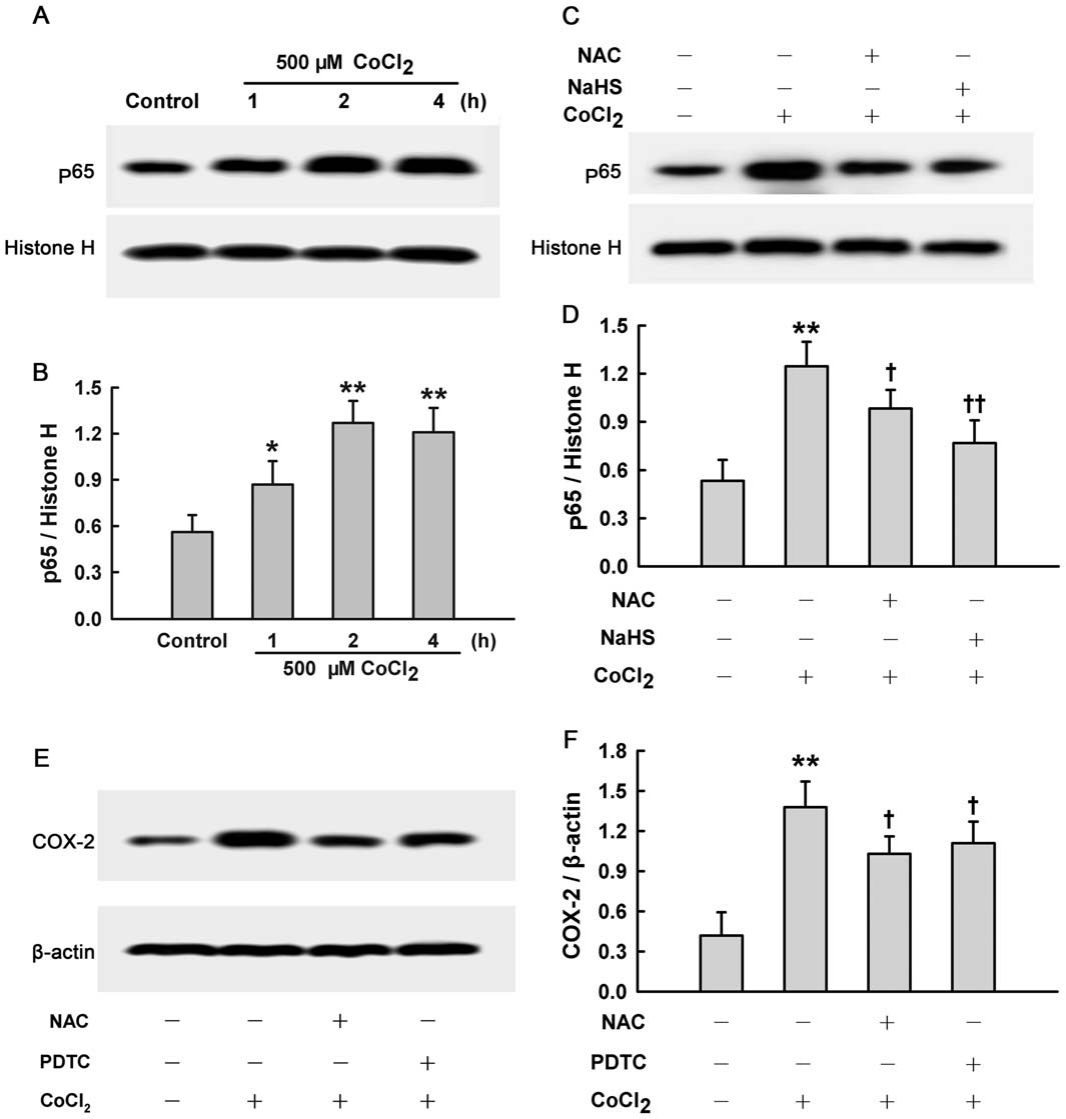
Effects of different treatments on CoCl_2_-induced intranuclear accumulation of NF-κB p65 subunit and overexpression of COX-2 in HaCaT cells. (**A**) HaCaT cells were treated with 500 µM CoCl_2_ for the indicated times. (**C**) HaCaT cells were pretreated with 400 µM NaHS for 30 min or 1000 µM NAC for 60 min followed by treatment with 500 µM CoCl_2_ for 2 h. Nuclear extract lysates were subjected to Western blot analysis using p65-specific antibody. (**E**) HaCaT cells were treated with 500 µM CoCl_2_ for 6 h in the absence or presence of pretreatment with 1000 µM NAC for 60 min or 10 µM PDTC for 30 min. Cell lysates were subjected to Western blot analysis using COX-2-specific antibody. Panels **B**, **D** and **F** show the intensity of the protein bands of typical experiments from A, C and E, respectively. Data were shown as the mean ± SE. ^*^ *P*<0.05, ^**^ *P*<0.01 compared with control group. ^+^ *P*<0.05, ^++^ *P*<0.01 compared with CoCl_2_ treatment group.

Exposure of HaCaT cells to 500 µM CoCl_2_ from 1 to 4 h significantly enhanced intranuclear NF-κB p65 subunit expression, the central step of NF-κB activation, compared with quiescent cells. This enhancement peaked at 2 h (Figure 6 A and B), indicating that CoCl_2_ exposure may evoke NF-κB activation. Before exposure to 500 µM CoCl_2_ for 2 h, pretreatment of HaCaT cells with 400 µM NaHS for 30 min (Figure 6 C and D) significantly inhibited NF-κB p65 subunit nuclear translocation. Importantly, pretreatment with PDTC (10 µM), a selective inhibitor of NF-κB, for 30 min before exposure to CoCl_2_ abrogated not only CoCl_2_-induced COX-2 overexpression (Figure 6 E and F), but also the secretions of IL-6 (Figure 5A), IL-8 (Figure 5B) and IL-1β (Figure 5C). In addition, pretreatment with PDTC also inhibited CoCl_2_-induced cytotoxicity (Figure 5D). These results suggest that the protection of H_2_S against inflammation and cytotoxicity caused by CoCl_2_ is partially associated with the inhibition of NF-κB activation in HaCaT cells.

### Inhibition of oxidative stress is involved in the protection of H_2_S against inflammation in CoCl_2_-treated HaCaT cells

Since antioxidation was implicated in the inhibitory effect of H_2_S on CoCl_2_-induced cytotoxicity, we further explored the role of antioxidation in H_2_S-triggered cytoprotection against CoCl_2_-induced inflammatory response. Similar to the anti-inflammatory effect of H_2_S, pretreatment of HaCaT cells with NAC suppressed secretions of IL-6 (Figure 5A), IL-8 (Figure 5B) and IL-1β (Figure 5C) induced by CoCl_2_. Further studies showed that pretreatment with NAC inhibited CoCl_2_-induced NF-κB p65 nuclear translocation (Figures 6C and D) and COX-2 overexpression (Figures 6E and F), so did NaHS pretreatment. The above results indicate that inhibition of oxidative stress may attribute to the protective effect of H_2_S against CoCl_2_-induced inflammatory response in HaCaT cells.

## Discussion

Hypoxia-induced dermatic injury occurs in many diseases, including pressure ulcers [14], diabetic ulcers [15] and venous ulcers [17]. Oxidative stress and inflammatory response are two key risk factors of these diseases. Anti-inflammatory medicines, such as steroid and non-steroid, have been used for the treatment of these diseases. However, these medicines have adverse side effects, such as increased susceptibility to infection, impaired glucose tolerance in diabetes, osteoporosis and gastrointestinal pain. It is therefore necessary to discover compounds with high efficacy and fewer side effects. H_2_S, an endogenous gaseous mediator, exerts various physiological and physiopathological effects *in vivo*, including anti-oxidative stress and anti-inflammatory response in heart, liver, kidney and other organs [5], [6], [7], [34]. We therefore hypothesize that H_2_S may confer protective effects against hypoxia-induced dermatic injury.

In the present study, chemical hypoxia was induced in the human skin keratinocytes (HaCaT cells) by exposure to CoCl_2_. This chemical hypoxic agent can take the place of ferrous ions in prolyl-4-hydroxylase (P4H), thereby causing a conformational change in the P4H protein which consequently leads to a hypoxic condition, characterized by intranuclear accumulation of hypoxia inducible factor 1 alpha (HIF-1α) [35], [36], [37]. Our results showed that exposure of HaCaT cells to CoCl_2_ led to cytotoxicity, evidenced by the decreased cell viability. To investigate whether H_2_S can protect HaCaT cells against CoCl_2_-induced cytotoxicity, HaCaT cells were pretreated with NaHS (a H_2_S donor) at concentrations ranging from 100 to 800 µM for 30 min before exposure to CoCl_2_. Interestingly, we found that pretreatment with NaHS significantly attenuated CoCl_2_-induced cytotoxicity in HaCaT cells. This anti-cytotoxic effect of H_2_S is similar to our previous results in H9c2 myocardial cells [38] and PC12 cells [39]. A recent study showed that NaHS (10 to 1000 µM) treatment for 20 min can protect human umbilical vein endothelial cells (HUVECs) and fibroblasts (3T3s) against ischemia-reperfusion (I/R)-induced apoptosis [40]. In addition, there are other studies reporting the protective effects of H_2_S in heart, liver, kidney and skin [5], [6], [7], [40], which provides a foundation for our current study. However, Gobbi *et al.* reported that H_2_S impairs keratinocyte growth and adhesion [41], which is opposite to our findings. In that study, NaHS concentrations were used ranging from 500 to 2000 µM and the treatment period ranging from 24 to 72 h. The difference between their results and ours may be due to differences in NaHS treatment mode.

Another important finding of this study was that H_2_S inhibited oxidative stress induced by CoCl_2_ in HaCaT cells. We used DCF staining followed by photofluorography to detect intracellular ROS level. We found that exposure to CoCl_2_ elicited a marked increase in ROS generation in HaCaT cells. The increased ROS production was significantly abrogated by pretreatment with NaHS. We speculated that one of the mechanisms underlying NaHS-induced ROS elimination may be associated with a direct chemical reaction with H_2_O_2_. Geng *et al.* reported that H_2_S directly scavenges superoxide anions and H_2_O_2_, and consequently eliminates ROS-induced MDA generation [9]. We also found that NAC, a ROS scavenger, afforded the similar protective effect of H_2_S. Another mechanism for the inhibition of oxidative stress by H_2_S may be associated with enhancing the endogenous antioxidative defense ability. For instance, GSH, a potent endogenous antioxidant, can eliminate ROS and be oxidized into glutathione disulfide [21]. In agreement with the previous evidence that NaHS reverses H_2_O_2_-impaired GSH production [23], our findings showed that H_2_S pretreatment effectively antagonized CoCl_2_-induced decrease in GSH level. Similarly, it has been shown that treatment with exogenous vitamin E suppresses pressure-induced skin lesions by inhibiting H_2_O_2_ generation and GSH loss [21]. Therefore, H_2_S pretreatment triggers a cytoprotective effect at least in part by its antioxidative function.

Inflammatory response is an important injury factor in hypoxia-induced dermatic ulcers. In this study, besides cytotoxicity and oxidative stress, chemical hypoxia induced inflammatory response, which was evidenced by increases in IL-6, IL-8 and IL-1β secretions. Importantly, we observed that pretreatment with NaHS significantly attenuated CoCl_2_-stimulated IL-6, IL-8 and IL-1β secretions from HaCaT cells, suggesting that H_2_S can protect HaCaT cells against chemical hypoxia-induced inflammatory response. In macrophages, H_2_S has been shown to attenuate lipopolysaccharide-induced formation of inflammatory mediators, including IL-6 [11]. In addition, H_2_S also inhibits IL-6 secretion of fibroblasts isolated from the synovial membrane of rheumatoid arthritis patients [42]. The above previous studies [11], [42] support our study.

COX-2 is a potent pro-inflammation mediator, which can promote the production of many inflammatory factors in various experiments. A previous study showed that COX-2 mRNA expression is upregulated in rat skin suffering from I/R lesion, and the selective inhibitor of COX-2, NS-398, abrogates nicotine aggravated-skin necrosis induced by I/R [43]. Our current study showed that exposure of HaCaT cells to CoCl_2_ elevated expression of COX-2 and induction of PGE_2_. The pretreatment with NaHS for 30 min suppressed CoCl_2_ stimulated- COX-2/PGE_2_ upregulation. Similar to the protective effect of H_2_S, inhibition of COX-2 by NS-398 attenuated not only CoCl_2_-induced cytotoxicity, but also the secretions of IL-6, IL-8 and IL-1β secretions. Our data revealed that COX-2/PGE_2_ pathway mediates CoCl_2_-induced inflammation and cytotoxicity, and that inhibition of COX-2/PGE_2_ pathway contributes to the protective effect of H_2_S. Similarly, Chi *et al.* reported that wogonin, which is derived from a traditional Chinese medicine Huang-Qin, reduces I/R-induced dermatic injury partly by inhibition of COX-2 [44]. In addition, in septic rat liver damage model, COX-2 inhibition by NS-398 can confer anti-inflammatory effects, increasing IL-10 secretion and decreasing IL-6 secretion [45]. Nevertheless, some research indicated that induction of COX-2/PGE2 mediates atorvastatin-induced cardioprotection [46]. COX-2 induction also contributes to delayed cardioprotection induced by H_2_S preconditioning in isolated rat cardiomyocytes [47]. Taken together, the reason for anti-inflammaroty or pro-inflammatory effect of COX-2 might be complicated. One explanation might be due to tissue-specific regulatory mechanisms. To elucidate this question, further studies are required.

NF-κB is an inducible transcription factor and can potently augment COX-2 expression [48]. The p65 protein is one of the most abundant subunits of NF-κB. Its nuclear translocation usually indicates the activation of NF-κB. An earlier study showed that CoCl_2_ promotes the translocation of NF-κB p65 subunit into nucleus and enhances its binding to a NF-κB consensus sequence in endothelial cells [49]. In accordance with the above report, we showed that exposure of HaCaT cells to CoCl_2_ led to the accumulation of intranuclear NF-κB p65 subunit, which was significantly repressed by pretreatment with NaHS. By inhibiting NF-κB, both H_2_S and PDCT (a selective inhibitor of NF-κB) attenuated CoCl_2_-induced overexpression of COX-2, oversecretion of inflammatory factors and cytotoxicity in HaCaT cells. These results are comparable with the previous findings that the H_2_S-releasing molecule, GYY4137, protects against lipopolysaccharide-induced endotoxic shock in the rat through inhibition of NF-κB upregulation [50]. Collectively, we provide new evidence that activation of NF-κB regulates COX-2-mediated inflammation and cytotoxicity, and that H_2_S protects against CoCl_2_-induced inflammation and cytotoxicity by inhibition of NF-κB/COX-2 pathway in HaCaT cells.

Numerous studies have demonstrated that ROS are important triggers to upregulate NF-κB activity and that antioxidants can be consequently applied to inhibit NF-κB activation [51], [52]. Our more recent study showed that ROS mediate CoCl_2_-induced activation of NF-κB/COX-2 pathway[26]. To determine whether antioxidation of H_2_S was involved in its inhibition of NF-κB/COX-2 pathway, we observed the effect of NAC, a ROS scavenger, on CoCl_2_-induced overexpressions of NF-κB p65 subunit and COX-2. Similar to H_2_S-induced inhibition of overexpressions of NF-κB p65 subunit and COX-2, pretreatment with NAC also obviously suppressed CoCl_2_-induced accumulation of intranuclear NF-κB p65 subunit and overexpression of COX-2. Further study also revealed that NAC significantly alleviated CoCl_2_-induced secretions of IL-6, IL-8 and IL-1β. These data indicated that H_2_S represses NF-κB/COX-2-mediated inflammation partially by its antioxidative effect.

In summary, the present study has for the first time demonstrated that H_2_S confers a cytoprotective effect against chemical hypoxia-induced cytotoxicity and inflammation through inhibition of the ROS-activated NF-κB/COX-2 signaling pathway in HaCaT cells. Our study provides new insights into the roles of H_2_S in attenuating hypoxia-induced dermatic injury. Modulation of endogenous H_2_S or exogenous administration of H_2_S may be a novel therapeutic strategy for dermatic injury induced by hypoxia.
